# Antibacterial effect of a new haemostatic agent on oral microorganisms

**DOI:** 10.4317/jced.50750

**Published:** 2012-07-01

**Authors:** Çağdaş Çinar, Mesut E. Odabaş, Gülçin Akca, Berrin Işik

**Affiliations:** 1 Assistant professor. Department of pediatric dentistry, faculty of dentistry, University of Gazi, Ankara, Turkey.; 2 MD, PhD. Department of basic medical sciences, microbiology laboratory, faculty of dentistry, University of Gazi, Ankara, Turkey.; 3 MD, Associate professor. Anesthesiology and reanimation specialist, department of anesthesiology and reanimation, faculty of medicine, University of Gazi, Ankara, Turkey.

## Abstract

Objective: The purpose of this study was to determine the antibacterial effect of a newly developed haemostatic agent Ankaferd Blood Stopper® (ABS) and Ferric Sulphate (FS) on various oral microorganisms. Study design: Bacterial strains were freshly incubated in their specific broth media. For each of the strains, 3 wells per each agent, with a 5 mm diameter were made under aseptic conditions in the specific agar media. Then they were filled with a test agents or 0.2% chlorhexidine digluconate (CHX) (control group). After 24h and 48h incubation periods, inhibition zones were measured. Results: ABS showed antibacterial effect on all test microorganisms except Lactobacillus acidophilus and Lactobacillus salivarius. Ferric sulphate and CHX have antibacterial effect on all microorganisms. When the test agents compared, the inhibition zones of the ABS were found smaller than the ferric sulphate and CHX. Conclusions: Although ferric sulphate and ABS have antibacterial effect, ferric sulphate had better antibacterial activity than ABS on oral microorganisms under in vitro condition. FS and ABS not only exhibit the haemostatic activity but also antimicrobial activity.

** Key words:**Ankaferd blood stopper, ferric sulphate, haemostatic agent, haemostasia, bleeding, bactericide.

## Introduction

Haemorrhage control is an essential factor in dental procedure for good visualization of the fields. Dentists per-form variety operative procedures such as pulpotomy, tooth extraction, biopsies, placement of endosseous implants, periradicular and periodontal surgery, which may require haemorrage control (1,2). The most widespread technique is to control bleeding by applying mechanical pressure to wound surface. The use of cotton balls or gauze pads risk leaving fibers in the surgical site. These fibers may elicit a foreign body reaction and delay healing (3). Another technique to control bleeding is the application of haemostatic agents(4). Various agents are used for hemorrhage control in dentistry.

Ferric sulphate (Fe2[SO4]3) has been used as a coagulative and haemostatic agent and it was first used in medicine in 1957. On contact with blood, the agglutination of blood proteins result from the reaction of blood with ferric and sulphate ions. Thus the capillary orifices have been obturate by bonded proteins (3).

Medical plants have been marketed as antibacterial agents and haemostatic agents for many years (5,6). Ankaferd Blood Stopper (ABS) (Ankaferd Sağlik Ürünleri A.Ş., Istanbul, Turkey) is a mixture of herbal medicine and it is recently developed to facilitate the rapid hemorrhage control in difficult clinical conditions (6). ABS ingredients a standard mixture of following plants: *Thymus vulgaris* (5.0g/100ml), *Glycyrrhiza glabra* (7.0g/100ml), *Vitis vinifera* (8.0g/100ml), *Alpinia officinarum* (7.0g/100ml) and *Urtica dioica* (6.0g/100ml). ABS stimulated rapid formation of a protein network and erythrocyte aggregation. The ABS-induced network formation is related to the function of blood proteins and red blood cells. The basic mechanism of action for ABS appears to be the formation of an encapsulated protein network that provides focal points for erythrocyte aggregation. ABS also interacts with fibrinogen and other blood proteins. Exposure to ABS seems to provide tissue oxygenation as well as a physiological haemostatic process without affecting any individual clotting factor. This unique mechanism of action provides ABS with an advantage (7). ABS has been safely used in patients to treat epistaxis (8), after tonsillectomy (9) or variceal bleeding(10). In addition, ABS has been used to control upper gastrointestinal bleeding (11). Moreover the levels of coagulation factors II, V, VIII, IX, X, XI, and XII were not affected by ABS; therefore, ABS might be used in patients with deficient primary haemostasis (12).

Many microorganism species have been detected in the oral cavity; some of these bacterial species can cause several diseases and complications. Infection of wounds is an important complication that impairs wound healing. A haemostatic agent should be bacteriostatic and/or bactericidal, when used in surgical wound. In this study, it was aimed to evaluate the potential antibacterial effect of ABS and FS under in vitro conditions.

## Material and Methods

Ankaferd Blood Stopper®, ferric sulphate and 0.2% chlorhexidine digluconate (control group) were screened for antimicrobial activity by using well-diffusion technique on agar plate.

Bacterial Strain and Culture Conditions

The antibacterial activity of each material was evaluated against *Staphylococcus aureus (S. aureus)* ATCC#25923, methicillin sensitive *S.aureus* (MSSA), methicillin resistant S. aureus (MRSA), *Enterococcus faecalis (E.faecalis)* (ATCC #29212), vancomycin resistant *E. faecalis* (VRE), *Candida albicans (C.albicans)*(ATCC#10231), *Candida albicans oral isolate*, *Porphyromonas gingivalis (P.gingivalis)* (ATCC#33277), *Lactobacillus acidophilus (L.acidophilus)* (ATCC#4356), *Lactobacillus salivarius (L.salivarius)*(ATCC#11741), *Streptococcus mutans (S.mutans)* (ATCC#25175), *Streptoccous sobrinus (S.sobrinus)* (ATCC#33478) and *Aggregatibacter actinomycetemcomitans (A.actinomycetemcomitans)* (ATCC#29523).

S.mutans and S.sobrinus, were cultured in 5 ml of brain heart infusion broth (BHIB, Oxoid, UK) at 37 ºC for 48 hours in an atmosphere of 5 % CO2. *E.faecalis* and *S.aureus* were cultured in 5 ml of brain heart infusion broth (BHIB, Oxoid, UK) at 37 ºC for 48 hours aerobically, *L.acidophilus* and *L.salivarius* were cultured at 37oC for 48 hours in 5 ml of MRS Broth (DeMan Rogosa and Sharpe Medium, Merck, Germany) in an atmosphere of 10% CO2. *C.albicans* was cultured in autoclaved-sterilized Sabourraud Dextrose Broth (SDB, Oxoid, UK) at 37oC for 48 hours under aerobic conditions. *A. actinomycetemcomitans* and *P.gingivalis* were cultured in auto-clave-sterilized trypticase soy broth (Oxoid, UK) supplemented sheep blood (50 ml/L), Vit K (1μg/ml) and hemin (5μg/ml) at 37oC for 4-5 days in an automatic anaerobic cabin (Electrotek AW400TG, UK) in the atmosphere consisted of 90% N2, 5% CO2, 5% H2. The last three ingredients were added to the main medium after filter-sterilized with 0.22μm millipore. All of the freshly grown bacterial suspensions in 5 ml of their specific broth media as described before were adjusted to 108 cfu/ml by using 0.5 Mc Farland test standard. For each of the strains, 3 wells per each agent with a 5 mm diameter were made under aseptic conditions in their specific agar media containing petri plates for each of the strains separately. Then the bacterial and fungal suspensions were spread on to their specific agar plates.

The wells were filled with 20?l ABS (Ankaferd® Sağlik Ürünleri A.Ş.) and Ferric sulhate (15.5%) (Astringedent®, Ultradent Products Inc., Salt Lake City, UT, USA). 0.2% chlorhexidine digluconate (CHX) was selected as a positive control group, because of its widespread clinical use; also it serves as a common point of reference for comparisons with other studies (13,14). Then, they were incubated on their specific media under standard microbiological conditions according to the growth properties as described before. After 48 hours the inhibition zones around the wells were measured with a digital caliper (Mitutoyo, SP, Brazil) for *P.gingivalis* and *A. actinomycetemcomitans* and 24 hours for the rest of the others, The measurements were repeated after 48 hours in the same manner. Tests were performed duplicate for all of these agents and materials. All inhibition zones were measured at 24 and 48 hours by one author.

The results were statistically analyzed using One-way analysis of variance and Tukey’s tests for the significant interrelation between the groups using SPSS 13.0 for Windows (SPSS Inc., Chicago, IL) with the level of statistical significance set at p<0.05.

## Results

The present study was designed to measure the direct inhibition of bacterial growth of 13 different bacteria. The inhibition degree of bacterial growth is shown in  Table 1. All values cited are expressed as means±SD. Ferric sulphate and CHX showed antibacterial effect against all microorganisms. No bacterial inhibition was detected after 24 - and 48 - hour incubation with ABS for *Lactobacillus acidophilus, Lactobacillus salivarius*. There was no statistical significance between ABS, ferric sulphate and CHX for *P.gingivalis* (P>0.05). CHX had the larger inhibition zone than ABS (p<0.05). In all tested microorganisms except *P.gingivalis*, a statistically significant difference was found between ABS and ferric sulphate (p<0.05) and, ABS showed minimum inhibition zone in 24 and 48 hours than CHX and ferric sulphate. Statistical analyses of tested haemostatic agents and control group is shown in  Table 2.

**Table 1:**
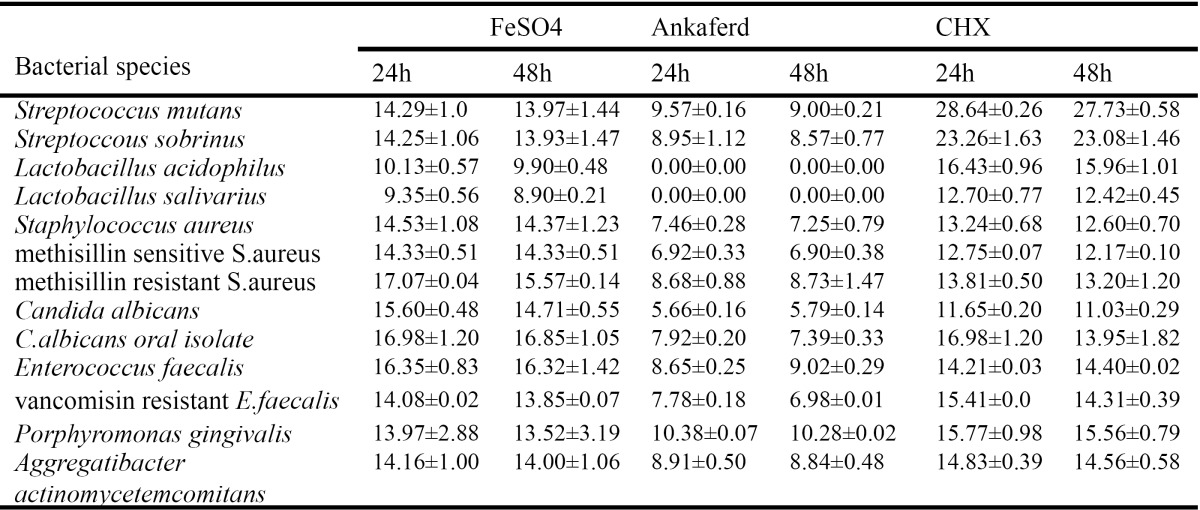
Antibacterial activity of tested haemostatic agents and control group, in millimeter inhibition (Mean ± SD) (n=3).

**Table 2:**
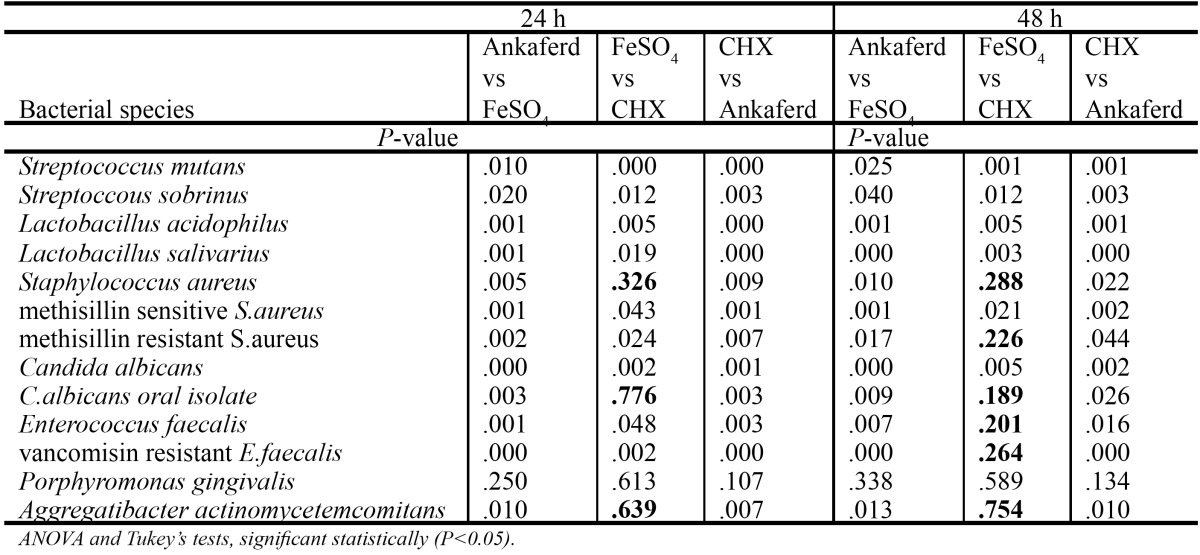
Statistical analyses of tested haemostatic agents and control group.

## Discussion

This article has described the antibacterial activity in vitro for newly developed haemostatic agent ABS. In this study, stains of *Streptococcus mutans, Streptococcus sobrinus, Lactobacillus acidophilus, Lactobacillus salivarius, Staphylococcus aureus*, Methicillin-resistant *staphylococcus aureus (MRSA)*, Methicillin-sensitive *Staphylococcus aureus (MSSA), Enterococcus faecalis*, Vancomycin-resistant *Enterococcus faecalis (VRE), Candida albicans, C.albicans oral isolate, Porphyromonas gingivalis, Aggregatibacter actinomycetemcomitans* were used, which present in the oral cavity. *S.mutans, S.sobrinus, Lactobacilli* species that are known as cariogenic microorganisms play a major role in dental plaque formation. S.aureus and E.faecalis are most commonly seen hospital infection of surgical wounds and infections associated with indwelling medical equipment. *S.aureus* is also a human pathogen, and it causes systemic infections such as wound infections, pneumonia, endocarditis, and septicemia. *Porphyromonas gingivalis* and *Aggregatibacter actinomycetemcomitans* play a major role in periodontitis and periodontal tissue destruction.

Agar well-diffusion test is widely used in in-vitro method of evaluation of antimicrobial activity of various chemicals. The size of inhibition zone depends on the solubility of the test material, time and temperature of incubation (15). According to the other studies (6,16), agar well diffusion test was used in the present methodology to verify the inhibition zone of the hemostatic agents, because it was clearly seen and reported that this chemical did not dissolved in broth media such as Mueller Hinton Broth.

Many haemostatic agents and procedures have been used for hemorrhage control such as ferric sulphate and newly developed ABS. ABS has been recommended in the management of external and intraoral hemorrhage such as pulpotomy, tooth extraction and flap operation in dentistry. One of the most commonly used haemostatic agents in dentistry is ferric sulphate. The agglutination of blood proteins result from the reaction of blood with ferric and sulphate ions. This ferric ion-protein complex mechanically seals the cut vessel and producing haemostasis. By forming plugs that occlude the capillary orifices, the protein complex also prevents the formation of blood clots. Ferric sulphate has antibacterial effect on all tested microorganisms in our study. This could be due to acidic pH(17) and cytotoxicity (3) of ferric sulphate solution. Lemon et al. (3) stated that although ferric sulfate was an effective haemostatic agent, healing was adversely affected. Jeansonne et al. (18) expressed that ferric sulphate provides effective haemostasis, and also irrigation with saline prior to closure of surgical defect, does not delay healing.

Although the ABS has antibacterial activity, the inhibition zones were found smaller than FS and CHX. Ferric sulphate showed inhibition zones against all of the microorganisms, ABS, unfortunately, did not show any in-hibition against both of the lactobacilli species. There was no statistical significance between ABS, ferric sulp-hate and CHX for *P.gingivalis* (P>0.05). The zones of bacterial growth of ferric sulphate and CHX became smaller depending on time. On the other hand, the zones of bacterial growth of ABS for some microorganisms became larger depending on time. However, these differences at 24- and 48- hour incubation for all tested materials and control group were insignificant to make inferences.

The effectiveness of ABS was due to the antimicrobial effect of several contents. Fukai et al. (19) stated that *Glycyrrhiza glabra* have antimicrobial activity against *S.aureus*, MSSA and MRSA. Lee et al. (20) explain that *Alpinia officinarum* has been used in Korea for several hundred years to treat various infectious diseases. Agnihotri and Vaidya(21) showed *Thymus vulgaris* has had antibacterial effect. Similar to our findings, Tasdelen-Fisgin et al.(6) reported that ABS has antibacterial activity against methicillin resistant *S.aureus* (MRSA), methicillin sensitive *S.aureus* (MSSA), and vancomycin resistant *E. faecalis* (VRE).

## Conclusions

In summary, a haemostatic agent should be bacteriostatic and/or bactericidal, when used in surgical wound. Based on the result of this study, FS had better antibacterial activity than ABS under in vitro conditions. The results of this study provide the information that FS and ABS not only exhibit the haemostatic activity but also antimicrobial activity. This may be an important finding in terms of wound healing.
